# Blue Honeysuckle (*Lonicera caerulea* L.) Polyphenol Extract Inhibits α-Glucosidase Activity and Modulates Glucose Transport in Caco-2 Cells

**DOI:** 10.3390/molecules31122146

**Published:** 2026-06-18

**Authors:** Fengfeng Li, Yao Wang, Huifang Shen, Xinting Shen, Fei Wang, Rui Zhao, Zhebin Li, Bo Li, Ye Zhou, Xinmiao Yao

**Affiliations:** 1 Food Processing Research Institute, Heilongjiang Academy of Agricultural Sciences, Harbin 150086, China; lff122ff@163.com (F.L.); wang1221yao1221@163.com (Y.W.); shenhuifang_1987@126.com (H.S.); sxt970901@126.com (X.S.); wangf2022822@163.com (F.W.); lilyamongthorns@163.com (R.Z.); lizhebin2010@163.com (Z.L.); blnky@163.com (B.L.); 2 Heilongjiang Province Key Laboratory of Food Processing, Harbin 150086, China; 3 Heilongjiang Province Engineering Research Center of Whole Grain Nutritious Food, Harbin 150086, China

**Keywords:** *Lonicera caerulea*, anthocyanins, cyanidin-3-O-glucoside, α-glucosidase inhibition, intestinal glucose transport, SGLT1, GLUT2, postprandial hyperglycemia

## Abstract

Blue honeysuckle (*Lonicera caerulea* L.) is a polyphenol-rich berry increasingly recognized as a functional food ingredient for postprandial glycemic management. However, it remains unclear whether its polyphenols can modulate intestinal glucose transport in addition to inhibiting carbohydrate-digesting enzymes. In this study, blue honeysuckle polyphenol extract (BHPE) was characterized by UPLC-QTOF-MS/MS, and its effects on α-glucosidase activity and intestinal glucose transport were evaluated using enzyme kinetics, fluorescence quenching, molecular docking, and differentiated Caco-2 monolayers. A total of 24 phenolic compounds were tentatively identified, with anthocyanins and chlorogenic acid derivatives as the major constituents. BHPE exhibited a mixed-type, static-quenching inhibition of α-glucosidase (IC_50_ = 75.05 μg/mL). Furthermore, molecular docking revealed that key constituents, including cyanidin-3-O-glucoside, chlorogenic acid, and proanthocyanidin B1, bind the enzyme via hydrogen bonding and hydrophobic interactions. In Caco-2 cell monolayers, BHPE reduced glucose transport by up to 51.56% under simulated postprandial conditions and coordinately downregulated *SGLT1* and *GLUT2* mRNA expression to 0.58- and 0.51-fold, respectively. These findings extend the bioactivity profile of blue honeysuckle polyphenols from enzyme-level inhibition to functional regulation at the intestinal epithelial barrier, highlighting their potential as multi-target natural ingredients for the attenuation of postprandial hyperglycemia.

## 1. Introduction

Postprandial hyperglycemia is a hallmark of type 2 diabetes mellitus (T2DM) and is closely associated with the development of various metabolic complications [1]. Postprandial glucose levels are largely determined by the intestinal digestion of dietary carbohydrates and the subsequent absorption of released glucose in the small intestine [2]. During carbohydrate digestion, α-amylase initiates starch hydrolysis by producing shorter maltoligosaccharides, whereas α-glucosidase catalyzes the final hydrolysis of oligosaccharides and disaccharides into absorbable monosaccharides [3]. The released glucose is then taken up and transported across intestinal epithelial cells mainly through sodium-dependent glucose cotransporter 1 (SGLT1) and glucose transporter 2 (GLUT2) [4]. Therefore, simultaneously targeting α-glucosidase and intestinal glucose transport represents a relevant strategy for reducing intestinal glucose availability.

Dietary polyphenols have attracted significant attention as natural modulators of postprandial glucose metabolism because of their multi-target activities [5]. At the digestive level, polyphenols can delay the conversion of dietary carbohydrates into absorbable glucose by inhibiting carbohydrate-hydrolyzing enzymes such as α-glucosidase [6]. Beyond enzyme inhibition, a growing body of evidence indicates that polyphenols can also interfere with intestinal glucose absorption by regulating glucose uptake, trans-epithelial transport, and the expression or activity of glucose transporters such as SGLT1 and GLUT2 [7]. These effects appear to be closely related to polyphenol structure and class, as anthocyanins, phenolic acids, proanthocyanidins, and other flavonoids may differentially influence glucose uptake, SGLT1/GLUT2-associated transport, transporter expression, or related signaling pathways [8,9,10]. However, digestive enzyme inhibition and epithelial glucose transport regulation are often investigated separately, and the relationship between phenolic composition and these two functional levels remains unclear for many polyphenol-rich food sources.

Blue honeysuckle (*Lonicera caerulea* L.), also known as haskap, is a polyphenol-rich berry that has attracted increasing interest because of its potential health-promoting properties [11]. Its phenolic profile is generally dominated by anthocyanins, especially cyanidin-3-O-glucoside, together with chlorogenic acid and other flavonoids [12]. Previous studies have mainly characterized its phenolic composition, antioxidant capacity, and α-glucosidase inhibitory activity [13,14]. However, whether blue honeysuckle polyphenol extract (BHPE) can regulate intestinal epithelial glucose uptake and transepithelial glucose transport remains insufficiently understood. Moreover, the relationship between its characteristic phenolic composition, α-glucosidase inhibition, and epithelial glucose transport regulation has not been systematically evaluated. This knowledge gap limits a comprehensive understanding of the potential role of BHPE in modulating intestinal glucose availability.

Therefore, this study aimed to provide an integrated in vitro evaluation of BHPE by combining phenolic profiling, α-glucosidase inhibition, and intestinal epithelial glucose transport assays. BHPE was characterized by UPLC-QTOF-MS/MS, and its interaction with α-glucosidase was investigated using inhibition kinetics, fluorescence quenching, and molecular docking of representative phenolic compounds. Differentiated Caco-2 monolayers were further used to evaluate cellular glucose uptake, transepithelial glucose transport under simulated preprandial and postprandial conditions, and the mRNA expression of *SGLT1* and *GLUT2*. This integrated design was used to clarify whether BHPE modulates intestinal glucose availability through both digestive enzyme inhibition and epithelial transport regulation. These findings may provide a scientific basis for the development of BHPE as a functional food ingredient for postprandial glycemic management.

## 2. Results and Discussion

### 2.1. Phenolic Profile of BHPE Characterized by UPLC-QTOF-MS/MS

**Figure 1:**
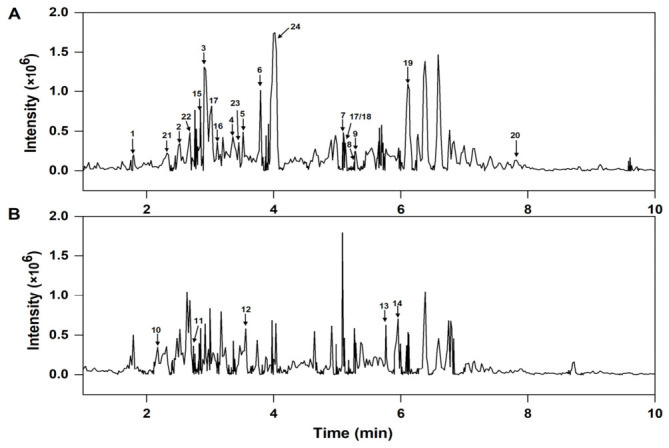
Total ion chromatograms (TICs) of phenolic compounds in the blue honeysuckle polyphenol extract (BHPE) acquired by UPLC-QTOF-MS/MS. (**A**) Positive ionization mode; (**B**) negative ionization mode. Peak numbers correspond to the compounds listed in Table 1.

The phenolic profile of BHPE was characterized by UPLC-QTOF-MS/MS in positive and negative ionization modes. As shown in Table 1 and Figure 1, a total of 24 phenolic compounds were tentatively identified, including 9 anthocyanins, 5 phenolic acids, and 10 flavonoids (mainly flavonols, flavanols and proanthocyanidins). These results indicate that BHPE contains a chemically diverse phenolic composition.

Anthocyanins were the main compounds detected in the positive ionization mode. Consistent with previous reports on blue honeysuckle berries, cyanidin derivatives were the predominant anthocyanins [15]. Compound 3 showed a precursor ion at *m*/*z* 449.1079 and a characteristic fragment ion at *m*/*z* 287.0548, corresponding to the cyanidin aglycone formed by the loss of a hexose moiety (162 Da). Therefore, this compound was tentatively identified as cyanidin-3-O-glucoside. Compound 1 presented a precursor ion at *m*/*z* 611.1603 and fragmented into *m*/*z* 449.1075 and *m*/*z* 287.0548 through loss of two hexose units, which was consistent with cyanidin-3,5-diglucoside. In addition, peonidin, pelargonidin, and delphinidin derivatives were also detected, further confirming the anthocyanin diversity of BHPE [13,16].

Phenolic acids were mainly detected in negative ionization mode, with chlorogenic acid and its isomers as the major constituents. Compounds 10, 11 and 12 showed a precursor ion at *m*/*z* 353.0878 and a characteristic fragment ion at *m*/*z* 191.057, corresponding to quinic acid generated by cleavage of the caffeoyl ester bond. Based on their retention times and reported fragmentation patterns, these compounds were tentatively assigned as neochlorogenic acid, chlorogenic acid, and cryptochlorogenic acid, respectively [17]. Di-caffeoylquinic acids (isochlorogenic acid B and C) were additionally identified at longer retention times with precursor ions at *m*/*z* 515.1193. Among flavonoids, several quercetins, kaempferol, and luteolin glycosides were tentatively characterized based on their diagnostic aglycone fragment ions at *m*/*z* 303, 287, and 287, respectively. Proanthocyanidin B1 was identified as a representative flavanol dimer, with a precursor ion at *m*/*z* 579.1479 and a fragment ion at *m*/*z* 291.0595 consistent with a catechin/epicatechin monomer unit. The presence of these structurally diverse flavonoids further attests to the compositional complexity of BHPE.

Overall, BHPE was mainly composed of anthocyanins, chlorogenic acid-related phenolic acids, and flavonoids, with cyanidin-3-O-glucoside, chlorogenic acid, and proanthocyanidin B1 as representative constituents. This phenolic profile is consistent with the characteristic composition of blue honeysuckle berries reported in the literature and provides a chemical basis for the subsequent evaluation of BHPE in α-glucosidase inhibition and intestinal glucose transport studies.

### 2.2. Inhibition of α-Glucosidase by BHPE

#### 2.2.1. Inhibitory Activity Analysis

**Figure 2:**
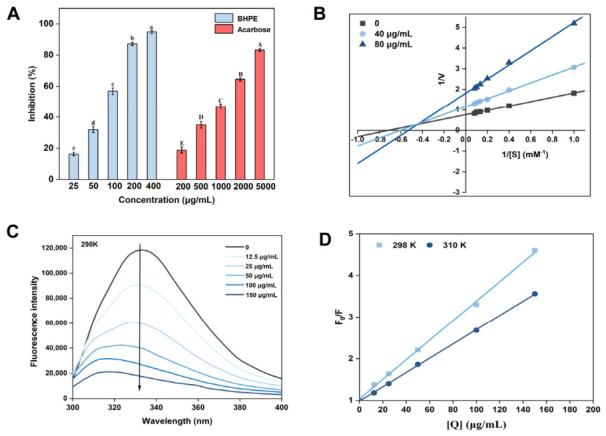
Inhibition of α-glucosidase by blue honeysuckle polyphenol extract (BHPE). (**A**) Inhibitory effects of BHPE and acarbose against α-glucosidase. (**B**) Lineweaver–Burk plots for α-glucosidase in the absence and presence of BHPE at 40 and 80 μg/mL. (**C**) Fluorescence emission spectra of α-glucosidase in the presence of BHPE at 0–150 μg/mL at 298 K. (**D**) Stern–Volmer plots for α-glucosidase at 298 K and 310 K. Different letters indicate significant differences (*p* < 0.05).

The inhibitory effect of BHPE on α-glucosidase activity is illustrated in Figure 2A. BHPE inhibited α-glucosidase in a clear concentration-dependent manner over the tested concentration range of 25–400 μg/mL, with the inhibition rate reaching 94.9% at 400 μg/mL. Based on regression analysis, the IC50 value of BHPE was calculated to be 75.05 μg/mL, indicating its strong inhibitory activity against α-glucosidase. In comparison, acarbose showed an IC_50_ value of 1013.68 μg/mL under the same conditions. The α-glucosidase inhibitory activity of BHPE is likely attributable to its rich phenolic composition, particularly the presence of anthocyanins, chlorogenic acid derivatives, and proanthocyanidins, which have been reported to interact with carbohydrate-hydrolyzing enzymes through hydrogen bonding, hydrophobic interactions, and other non-covalent forces. Similar inhibitory effects of anthocyanin-rich blue honeysuckle extracts against α-glucosidase have also been reported by Zhang, et al. [18].

#### 2.2.2. Enzyme Kinetics Analysis

Enzyme kinetic analysis was performed to further clarify the inhibition pattern of BHPE against α-glucosidase. As shown in Figure 2B, Lineweaver–Burk plots obtained at different BHPE concentrations intersected in the second quadrant rather than on the x- or y-axis, indicating a mixed-type inhibition pattern. With increasing BHPE concentration, V_max_ decreased, while K_m_ increased, suggesting that BHPE could interact with both the free enzyme and the enzyme–substrate complex. This mixed-type inhibition pattern is consistent with the complex and heterogeneous phenolic composition of BHPE, as structurally distinct phenolic constituents may engage different binding regions of α-glucosidase. Similar mixed-type kinetics have been reported for other polyphenol-rich plant extracts [19,20].

#### 2.2.3. Fluorescence Quenching Analysis

Fluorescence spectroscopy was used to investigate the interaction between BHPE and α-glucosidase. As shown in Figure 2C, α-glucosidase exhibited a characteristic intrinsic fluorescence emission peak at approximately 330 nm when excited at 280 nm. With increasing BHPE concentration from 12.5 to 150 μg/mL, the fluorescence intensity of α-glucosidase decreased progressively, indicating that BHPE quenched the intrinsic fluorescence of the enzyme. Meanwhile, the maximum emission wavelength shifted from 330 to 320 nm, suggesting that the interaction with BHPE altered the microenvironment around the fluorescent amino acid residues of α-glucosidase and made it relatively less polar and more hydrophobic [21].

To clarify the quenching mechanism, Stern–Volmer plots were constructed at 298 and 310 K (Figure 2D, Table 2). The Stern–Volmer quenching constant (K_sv_) decreased from 2.34 × 10^−2^ to 1.71 × 10^−2^ mL/μg as temperature increased, and the quenching rate constant (K_q_) decreased from 2.34 × 10^6^ to 1.71 × 10^6^ mL/(μg·s). The decrease in quenching efficiency with increasing temperature suggests that the fluorescence quenching of α-glucosidase by BHPE mainly followed a static quenching mechanism, which is associated with the formation of a BHPE–α-glucosidase complex rather than dynamic collision [22]. Double-logarithmic analysis revealed approximately one binding site per enzyme molecule (*n* ≈ 1.0) at both temperatures. The binding constant (K_a_) increased from 27.40 to 78.32 mL/μg with increasing temperature, implying that hydrophobic interactions may contribute to the binding process, as such interactions are often enhanced at higher temperatures. Together, these results indicate that BHPE interacted with α-glucosidase and modified the microenvironment around its fluorescent amino acid residues [23].

#### 2.2.4. Molecular Docking Analysis

**Figure 3:**
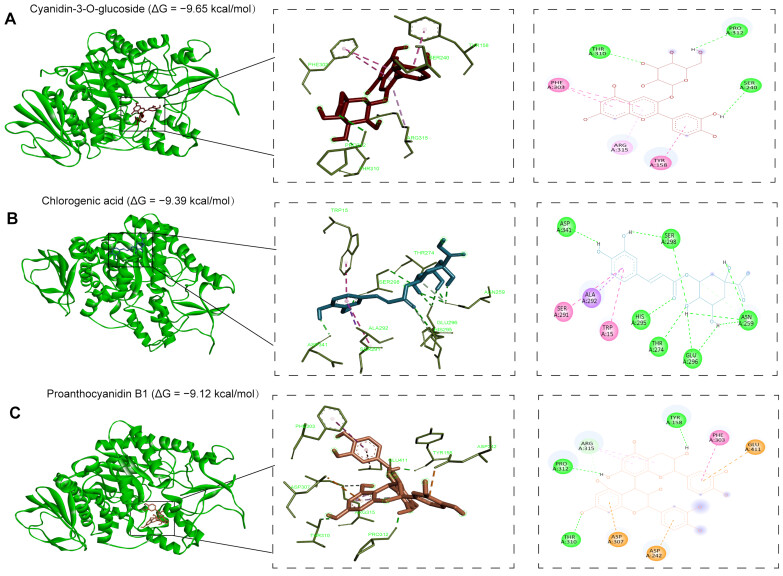
Molecular docking of three representative phenolic constituents of BHPE with α-glucosidase. (**A**) Cyanidin-3-O-glucoside (−9.65 kcal/mol) (**B**) Chlorogenic acid (−9.39 kcal/mol) (**C**) Proanthocyanidin B1 (−9.12 kcal/mol). For each ligand, the left panel shows the binding pose in the active-site cavity, the middle panel shows key residues in the binding pocket, and the right panel shows the 2D interaction map. Dashed lines indicate the predicted non-covalent interactions between ligands and amino acid residues.

Molecular docking was performed to predict binding modes between α-glucosidase and representative phenolic constituents of BHPE, including cyanidin-3-O-glucoside (C3G), chlorogenic acid, and proanthocyanidin B1 (Figure 3) [24]. These compounds were selected to represent the main phenolic classes in BHPE, namely anthocyanins, phenolic acids, and flavanols/proanthocyanidins. All three ligands showed favorable binding affinities toward α-glucosidase, with binding energies lower than −9.0 kcal/mol, indicating stable interactions with the enzyme active site or its vicinity.

As shown in Figure 3A, C3G exhibited the lowest binding free energy (−9.65 kcal/mol) among the three compounds and was predicted to interact with residues THR310, PRO312, SER240, TYR158, PHE303, and ARG315 through hydrogen bonding and hydrophobic contacts. Chlorogenic acid showed a binding free energy of −9.39 kcal/mol, with its binding conformation stabilized by hydrogen bonds with ASP341, SER298, HIS295, GLU296, ASN259, and THR274 (Figure 3B). Proanthocyanidin B1 displayed a binding free energy of −9.12 kcal/mol and was predicted to interact with residues including PRO312, TYR158, and THR310 (Figure 3C). These different binding conformations indicate that phenolic compounds from different structural classes may interact with α-glucosidase in distinct ways, mainly through hydrogen bonding and other non-covalent interactions [25]. This may partly support the mixed-type inhibition pattern observed in the enzyme kinetic analysis.

### 2.3. Effects of BHPE on Intestinal Glucose Transport

#### 2.3.1. Cell Viability Analysis

**Figure 4:**
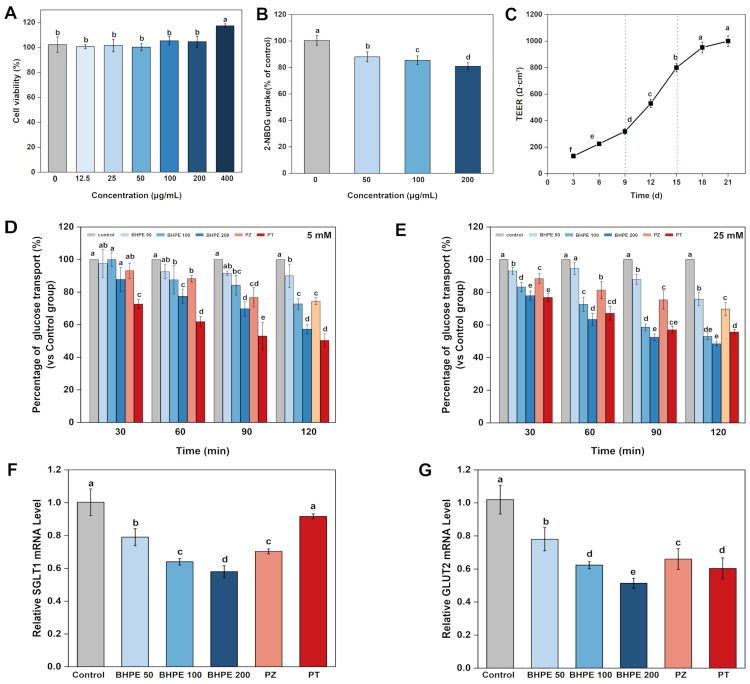
Effects of BHPE on Caco-2 cell viability, glucose uptake, monolayer integrity, transepithelial glucose transport, and relative mRNA expression. (**A**) Cell viability. (**B**) 2-NBDG uptake. (**C**) TEER during monolayer formation. (**D**,**E**) Relative glucose transport at 5 mM and 25 mM glucose, respectively. (**F**) *SGLT1* and (**G**) *GLUT2*. PZ (phlorizin) and PT (phloretin) were used as SGLT1 and GLUT2 inhibitors. Different letters indicate significant differences (*p* < 0.05).

Before evaluating the effects of BHPE on glucose uptake and transport, its potential cytotoxicity toward Caco-2 cells was assessed using the MTT assay. As shown in Figure 4A, BHPE did not reduce cell viability within the tested range of 12.5–400 μg/mL. Cell viability remained above 100% in all BHPE-treated groups, ranging from 110.6% to 122.0%, with a slight but significant increase observed at 400 μg/mL (*p* < 0.05). This increase may reflect enhanced mitochondrial metabolic activity rather than increased cell number. Therefore, 50, 100, and 200 μg/mL were selected as non-cytotoxic working concentrations with a clear low-to-high dose range for subsequent glucose uptake and transepithelial transport experiments.

#### 2.3.2. Inhibition of Cellular Glucose Uptake

The effect of BHPE on cellular glucose uptake in Caco-2 cells was evaluated using the fluorescent glucose analog 2-NBDG [26,27]. As shown in Figure 4B, BHPE reduced 2-NBDG uptake in a concentration-dependent manner. Compared with the control group, treatment with BHPE at 50, 100, and 200 μg/mL decreased 2-NBDG uptake by 11.21%, 18.79%, and 30.29%, respectively (*p* < 0.05). These results indicate that BHPE suppressed glucose uptake by Caco-2 cells under the tested conditions. Previous studies have also reported that anthocyanin-rich extracts and cyanidin derivatives can inhibit glucose uptake in intestinal cell models [8]. Given that cyanidin-3-O-glucoside is one of the predominant anthocyanins in BHPE, it may partly contribute to the observed inhibition of cellular glucose uptake. However, considering the complex phenolic composition of BHPE, this effect is likely attributable to the additive or synergistic effects of its various phenolic components.

#### 2.3.3. Integrity Assessment of Caco-2 Cell Monolayers

Establishing an intact and differentiated Caco-2 monolayer is essential for evaluating transepithelial glucose transport [28]. TEER values were monitored during the 21-day culture period to assess monolayer integrity (Figure 4C). TEER values increased gradually during the initial culture stage and rose markedly after day 9, reflecting progressive tight junction maturation. By day 21, the TEER value reached approximately 1015 Ω·cm^2^, which was well above the commonly accepted threshold of 400 Ω·cm^2^ for transport experiments [29]. These results confirm that the Caco-2 monolayers developed sufficient barrier integrity for use in subsequent glucose transport assays.

#### 2.3.4. Inhibition of Glucose Transport in Caco-2 Monolayers

To evaluate the effect of BHPE on intestinal glucose transport, transepithelial glucose transport across Caco-2 monolayers was measured for 120 min under simulated preprandial (5.0 mM glucose) and postprandial (25.0 mM glucose) conditions (Figure 4D,E). PZ and PT were used as reference inhibitors of SGLT1 and GLUT2, respectively.

Under preprandial conditions, BHPE inhibited glucose transport in a concentration- and time-dependent manner (Figure 4D). At 30 min, significant inhibition was observed only at 200 μg/mL (17.86%), which was higher than that of PT (7.54%) but lower than that of PZ (27.34%). At 120 min, the inhibition rate of 200 μg/mL BHPE increased to 42.78%, which was comparable to that of PZ (49.59%) and higher than that of PT (25.61%). Because SGLT1 is generally considered the major transporter responsible for glucose uptake under relatively low luminal glucose conditions, these results suggest that BHPE may partially interfere with SGLT1-associated glucose transport under the preprandial condition.

Under postprandial conditions, the inhibitory effect of BHPE was more pronounced (Figure 4E). At 120 min, BHPE at 50, 100, and 200 μg/mL inhibited glucose transport by 24.37%, 46.97%, and 51.56%, respectively. Under these high-glucose conditions, PT showed a stronger inhibitory effect than PZ, with inhibition rates of 44.34% and 30.25%, respectively, which is consistent with the increased contribution of GLUT2-mediated transport when luminal glucose concentration is elevated. Notably, 200 μg/mL BHPE showed a higher inhibitory effect than either PZ or PT alone, suggesting that BHPE may affect multiple glucose transport-related pathways rather than acting exclusively on a single transporter.

Collectively, these results indicate that BHPE suppressed transepithelial glucose transport across Caco-2 monolayers under both preprandial and postprandial glucose conditions. The different inhibition patterns observed at 5.0 and 25.0 mM glucose suggest that BHPE may modulate both SGLT1- and GLUT2-associated transport processes, depending on luminal glucose availability [30]. This finding is consistent with previous reports that polyphenol-rich extracts can reduce intestinal glucose transport through multiple mechanisms, including modulation of glucose transporter activity and expression [5,7,9]. However, because PZ and PT were used only as reference inhibitors, the precise contribution of each transporter to the overall inhibitory effect of BHPE requires further confirmation. Therefore, glucose transporter-related gene expression was further examined to provide additional mechanistic evidence.

#### 2.3.5. Modulation of *SGLT1* and *GLUT2* Gene Expression

To further examine transporter-related regulation, the mRNA expression levels of *SGLT1* and *GLUT2* were measured by RT-qPCR in Caco-2 monolayers under simulated postprandial high-glucose conditions (25.0 mM glucose). As shown in Figure 4F,G, BHPE significantly downregulated the mRNA expression of *SGLT1* and *GLUT2* in a concentration-dependent manner (*p* < 0.05). Treatment with BHPE at 50–200 μg/mL reduced *SGLT1* expression to 0.79–0.58-fold and *GLUT2* expression to 0.78–0.51-fold relative to the control group. At 200 μg/mL, BHPE reduced *SGLT1* and *GLUT2* expression to 0.58- and 0.51-fold of the control, respectively, which were lower than those observed in the corresponding reference inhibitor groups, namely PZ for *SGLT1* and PT for *GLUT2*. These results suggest that the inhibitory effect of BHPE on transepithelial glucose transport under high-glucose conditions may be partly associated with the downregulation of glucose transporter-related gene expression.

These findings are consistent with previous studies showing that polyphenol-rich extracts can regulate glucose transporter expression in intestinal cell models. For example, Mieres-Castro, Theoduloz, Sus, Burgos-Edwards, Schmeda-Hirschmann, Frank and Jiménez-Aspee [10] reported that *Gaultheria* berry extracts reduced *SGLT1* and *GLUT2* mRNA levels in Caco-2 cells. Similarly, mulberry leaf polyphenols have been shown to attenuate glucose absorption in Caco-2 cells by suppressing disaccharidase activity and modulating the SGLT1/GLUT2-related transport axis [9]. In the present study, the coordinated downregulation of *SGLT1* and *GLUT2* further supports the view that BHPE reduces intestinal glucose transport through transporter-related regulation. However, the present analysis was limited to mRNA expression. Therefore, further studies are required to determine whether these transcriptional changes are accompanied by corresponding alterations in SGLT1 and GLUT2 protein expression, membrane localization, and transporter activity. In addition, the upstream signaling pathways involved in BHPE-mediated regulation of glucose transport, such as AMPK-related pathways, remain to be clarified.

From an application perspective, BHPE may be suitable for incorporation into starch-rich foods, such as bread, noodles, pasta, and other cereal-based products, where regulation of carbohydrate digestion and intestinal glucose absorption is particularly relevant. Its potential benefit may arise from the combined regulation of α-glucosidase-mediated glucose release and epithelial glucose transport. However, practical application requires further evaluation of the processing and storage stability of anthocyanins and other phenolics, as well as their effects on sensory attributes and interactions with starch/protein food matrices. Future studies should therefore assess the matrix compatibility, sensory acceptability, phenolic bioaccessibility, and postprandial glycemic responses of BHPE-fortified starch-rich foods.

## 3. Materials and Methods

### 3.1. Materials and Reagents

Fresh blue honeysuckle berries were harvested at commercial maturity in July 2024 from Huma County, Daxing’anling Region, China (51°44′ N, 126°39′ E). The berries had a soluble solids content of 9.8 °Brix and a titratable acidity of 3.87 g/100 g. After harvest, the berries were immediately stored at −80 °C until use.

α-Glucosidase, *p*-nitrophenyl-α-D-glucopyranoside (pNPG), acarbose, phlorizin (PZ), and phloretin (PT) were purchased from Yuanye Biotechnology Co., Ltd. (Shanghai, China). Caco-2 cells were obtained from the Cell Bank of the Chinese Academy of Sciences (Shanghai, China). RPMI 1640 medium, fetal bovine serum (FBS) and phosphate-buffered saline (PBS) were supplied by HyClone Laboratories Inc. (Logan, UT, USA). A glucose assay kit was provided by Solarbio Science Technology Co., Ltd. (Beijing, China). The fluorescent glucose analog 2-NBDG was purchased from APExBIO (Beijing, China). All other chemicals and solvents were of analytical grade unless stated otherwise.

### 3.2. Preparation and Purification of Blue Honeysuckle Polyphenol Extract

Fresh berries were homogenized and extracted with 80% ethanol (*v*/*v*) at a solid-to-liquid ratio of 1:10 (*w*/*v*). Ultrasound-assisted extraction was performed at 40 kHz and 120 W for 90 min. The extract was centrifuged at 8000× *g* for 15 min, and the supernatant was collected. Ethanol was then removed using a rotary evaporator at 40 °C. The crude extract was further purified using an AB-8 macroporous resin column. After adsorption for 12 h, the column was washed with distilled water to remove water-soluble impurities, including sugars and organic acids. The adsorbed polyphenols were then eluted with 80% ethanol (*v*/*v*). The eluate was concentrated under reduced pressure and freeze-dried to obtain purified blue honeysuckle polyphenol extract (BHPE). The BHPE powder was stored at −20 °C in the dark until further analysis.

Total phenolic content (TPC) of BHPE was determined by the Folin–Ciocalteu method [31]. Total anthocyanin content (TAC) was measured using the pH differential method [32]. The TPC and TAC of BHPE were 444.11 ± 13.36 mg GAE/g DW and 184.39 ± 6.98 mg C3G/g DW, respectively.

### 3.3. Characterization of Phenolic Compounds by UPLC-QTOF-MS/MS

Phenolic compounds in BHPE were qualitatively characterized by UPLC-QTOF-MS/MS according to Fan, et al. [33] with minor modifications. Chromatographic separation was performed on an ACQUITY UPLC BEH C18 column (2.1 × 100 mm, 1.7 μm; Waters, Milford, MA, USA) at 30 °C. The mobile phase comprised 0.1% formic acid in water (phase A) and 0.1% formic acid in acetonitrile (phase B) at a flow rate of 0.3 mL/min with an injection volume of 5 μL. Gradient elution was programmed as follows: 0–20 min, 10–15% B; 20–25 min, 15–10% B.

Mass spectrometric analysis was performed on a TripleTOF 6600 system (SCIEX, Framingham, MA, USA) equipped with an electrospray ionization (ESI) source operated in both positive and negative ion modes. Full-scan data were acquired over an *m*/*z* range of 100–1500. Key MS parameters were as follows: ion spray voltage, +5500/−4500 V; source temperature, 550 °C; curtain gas, 35 psi; nebulizer gas (Gas 1), 50 psi; and heater gas (Gas 2), 50 psi. Data processing was performed using PeakView v2.2 and MasterView v1.1 software (SCIEX). Phenolic compounds were tentatively identified by comparing accurate mass, MS/MS fragmentation patterns, online databases, and previously reported literature.

### 3.4. α-Glucosidase Inhibition Assay

#### 3.4.1. Inhibitory Activity

The α-glucosidase inhibitory activity of BHPE was assessed following the method of Podsedek, et al. [34] with minor modifications. Briefly, 25 μL of α-glucosidase solution (0.5 U/mL, pH 6.8) was mixed with 25 μL of BHPE at different concentrations (25–400 μg/mL) and 25 μL of PBS in a 96-well microplate. After pre-incubation at 37 °C for 15 min, the reaction was initiated by adding 50 μL of pNPG solution (5 mM). The mixture was further incubated at 37 °C for 30 min, and the reaction was terminated by adding 100 μL of Na_2_CO_3_ (1 M). Absorbance was measured at 405 nm using a microplate reader. Acarbose was used as the positive control. The inhibition rate was calculated as follows:

(1) $$Inhibition\left(\%\right)=\left[1-\frac{\left(A_{sample}-A_{sample\;blank}\right)}{\left(A_{control}-A_{control\;blank}\right)}\right]\times 100\%$$

where A_sample_ is the absorbance of the reaction system containing BHPE and enzyme; A_sample blank_ is the absorbance of the system containing BHPE without enzyme; A_control_ is the absorbance of the system containing enzyme without BHPE; and A_control blank_ is the absorbance of the system without both BHPE and enzyme.

#### 3.4.2. Inhibition Kinetics

The inhibition type of BHPE against α-glucosidase was determined using Lineweaver–Burk double-reciprocal plots. Assays were performed as described in Section 3.4.1, with BHPE at 0, 40, and 80 μg/mL and pNPG at 0.5, 2.5, 5.0, 7.5, and 10 mM. The enzyme concentration was fixed at 0.5 U/mL. Initial reaction rate was calculated from the absorbance at 405 nm. Kinetic parameters *K*_m_ and *V*_max_ were calculated from the Lineweaver–Burk equation:

(2) $$\frac{1}{V}=\frac{K_{m}}{V_{max}}\times \frac{1}{\left[S\right]}+\frac{1}{V_{max}}$$

where V is the initial reaction rate, and V_max_ is the maximum reaction rate, K_m_ is the Michaelis–Menten constant, and [S] the substrate concentration.

#### 3.4.3. Fluorescence Quenching Assay

Fluorescence spectra were recorded using an RF-6000 fluorescence spectrophotometer (Shimadzu, Kyoto, Japan) following Tang, et al. [35]. Briefly, α-glucosidase (1 U/mL) was mixed with BHPE at 0, 12.5, 25, 50, 100, and 150 μg/mL and incubated in the dark for 5 min at 298 K or 310 K. Emission spectra were recorded from 300 to 400 nm at an excitation wavelength of 280 nm. The fluorescence quenching mechanism was analyzed using the Stern–Volmer equation:

(3) $$\frac{F_{0}}{F}=1+K_{sv}\left[Q\right]=1+K_{q}\tau_{0}\left[Q\right]$$

The binding constant and the number of binding sites were estimated using the double-logarithm equation:

(4) $$\log\left(\frac{F_{0}-F}{F}\right)=\log K_{a}+n\log\left[Q\right]$$

where F_0_ and F are the fluorescence intensities of α-glucosidase in the absence and presence of the BHPE, respectively; [Q] is the concentration of BHPE; K_SV_ is the Stern–Volmer quenching constant; K_q_ is the bimolecular quenching constant; τ_0_ is the average fluorophore lifetime (1 × 10^−8^ s); K_a_ is the binding constant; and *n* is the number of binding sites.

#### 3.4.4. Molecular Docking

Molecular docking was performed to predict the binding interactions between α-glucosidase and representative phenolic compounds of BHPE. Cyanidin-3-O-glucoside, chlorogenic acid, and proanthocyanidin B1 were selected as representative ligands based on the UPLC-QTOF-MS/MS results. Three-dimensional ligand structures were retrieved from PubChem in SDF format and geometry-optimized using ChemBio3D Ultra 14.0 (RMS gradient < 0.001). The crystal structure of α-glucosidase (PDB ID: 3A4A) was downloaded from the RCSB Protein Data Bank and prepared using PyMOL v2.3.0 by removing water molecules, co-crystallized ligands, and metal ions. Docking was conducted using AutoDock Vina v1.1.2, and the pose with the lowest binding affinity score was selected for interaction analysis. Binding interactions were visualized using PyMOL v2.3.0 and LigPlot+ v2.2.5.

### 3.5. Caco-2 Cell Model Experiments

#### 3.5.1. Cell Culture

Caco-2 cells were cultured in RPMI 1640 medium supplemented with 10% FBS (*v*/*v*) and 1% penicillin–streptomycin solution (*v*/*v*). Cells were cultured at 37 °C in a 5% CO_2_ atmosphere. When cell confluence reached 70–80%, they were passaged using 0.25% trypsin-EDTA. To ensure experimental consistency and phenotypic stability, all experiments in this study utilized cells at the 20th to 30th passage.

#### 3.5.2. Cell Viability Assay

The cytotoxicity of BHPE on Caco-2 cells was evaluated using the MTT assay. Briefly, cells were seeded at 1 × 10^4^ cells/well in 96-well plate and allowed to adhere for 24 h. The medium was then replaced with fresh medium containing different concentrations of BHPE ranging from 12.5 to 400 μg/mL. After 24 h of treatment, 10 μL MTT solution was added to each well, followed by a further 4 h of incubation at 37 °C. The supernatant was carefully removed, and the resulting formazan crystals were dissolved in 150 μL of DMSO. The absorbance was measured at 490 nm using a microplate reader. Cell viability was calculated using Equation:

(5) $$Cell\;viability(\%)=\frac{{OD}_{sample}-{OD}_{blank}}{{OD}_{control}-{OD}_{blank}}\times 100$$

where OD_sample_ is the absorbance of cells treated with BHPE; OD_control_ the absorbance of untreated cells (medium only); OD_blank_ the absorbance of the wells without cells.

#### 3.5.3. Measurement of Glucose Uptake Using 2-NBDG Fluorescence

Cellular glucose uptake was assessed using the fluorescent glucose analog 2-NBDG [36]. Caco-2 cells were seeded at 1 × 10^4^ cells/mL in black 96-well microplates with clear bottoms and allowed to adhere for 24 h. Cells were then treated with serum-free medium containing BHPE at 0, 50, 100, and 200 μg/mL for 24 h. Prior to the uptake assay, cells were washed twice with pre-warmed PBS and incubated in PBS at 37 °C for 30 min. Subsequently, 100 µL of 2-NBDG (100 μM) was added to each well and incubated at 37 °C in the dark for 30 min. The reaction was terminated by washing the cells three times with ice-cold PBS to remove extracellular 2-NBDG and terminate glucose uptake. Intracellular fluorescence intensity was measured using a fluorescence microplate reader (excitation/emission: 485/535 nm). Glucose uptake was expressed as a percentage of the control group.

#### 3.5.4. Establishment and Integrity Assessment of Caco-2 Cell Monolayers

Caco-2 cells were seeded at 1 × 10^5^ cells/mL onto polycarbonate Transwell insert filters (12-well, 0.4 µm pore size, 1.12 cm^2^ growth area, Corning Inc., Corning, NY, USA). The apical (AP) and basolateral (BL) chambers were filled with 0.5 and 1.5 mL of complete culture medium, respectively. Cells were cultured for 21 days to allow monolayer formation and differentiation. The medium was refreshed every other day for the first week and daily thereafter. Monolayer integrity was monitored by transepithelial electrical resistance (TEER) using a Millicell-ERS volt-ohmmeter (Millipore, Bedford, MA, USA). TEER values were measured on days 3, 6, 9, 12, 15, 18, and 21. The final TEER value was calculated using the following formula:

(6) $$TEER\left(\Omega \cdot {\mathrm{cm}}^{2}\right)=\left(R_{sample}-R_{blank}\right)\times 1.12$$

where R_sample_ is the measured resistance of the cell monolayer, R_blank_ is the resistance of the blank insert.

#### 3.5.5. Measurement of Glucose Transport Across Caco-2 Monolayers

Glucose transport was evaluated under simulated preprandial (5.0 mM) and postprandial (25.0 mM) glucose conditions according to Liu, et al. [37]. Before the transport assay, the monolayers were washed twice with pre-warmed Hank’s Balanced Salt Solution (HBSS, pH 7.4, containing Ca^2+^ and Mg^2+^) and equilibrated at 37 °C for 30 min. The BL chamber was filled with 1.5 mL of HBSS, while the AP chamber was filled with 0.5 mL of HBSS containing glucose (5.0 or 25.0 mM) and BHPE (0, 50, 100, or 200 μg/mL). At 30, 60, 90, and 120 min, 100 μL aliquots were collected from the BL chamber and replaced with an equal volume of fresh pre-warmed HBSS. Glucose concentrations in BL samples were determined using a GOD-POD glucose assay kit (Solarbio Science Technology Co., Ltd., Beijing, China). Phlorizin (PZ, 1 mM) and phloretin (PT, 1 mM) were used as reference inhibitors of SGLT1 and GLUT2, respectively [9].

#### 3.5.6. Quantitative Real-Time PCR (qRT-PCR) Analysis

The effects of BHPE on glucose transporter gene expression were evaluated by qRT-PCR. After monolayer formation, Caco-2 monolayers were apically treated with serum-free medium containing 25.0 mM glucose and BHPE at 0, 50, 100, or 200 μg/mL for 24 h. PZ (1 mM) and PT (1 mM) were used as reference inhibitor treatments. After treatment, cells were washed three times with ice-cold PBS. Total RNA was extracted using TRIzol reagent (Invitrogen, Carlsbad, CA, USA) according to the manufacturer’s instructions. RNA concentration and purity were assessed using a NanoDrop 2000 spectrophotometer (Thermo Fisher Scientific, Waltham, MA, USA), and samples with an A_260_/A_280_ ratio between 1.8 and 2.0 were used for subsequent analysis. The RNA was reverse-transcribed into cDNA using a HyperScript First-Strand cDNA Synthesis Kit (APExBIO, Beijing, China). Quantitative PCR was performed using HotStart^TM^ 2 × SYBR Green Master Mix on an Applied Biosystems StepOnePlus real-time PCR system (Applied Biosystems, Foster City, CA, USA). Relative mRNA expression levels of *SGLT1* and *GLUT2* were normalized to the GAPDH and calculated using the 2^−ΔΔCT^ method. The primer sequences in this study are listed in Table S1.

### 3.6. Statistical Analysis

All experiments were performed in triplicate, and data are expressed as the means ± standard deviation (SD). Statistical differences were analyzed using one-way analysis of variance (ANOVA) followed by Duncan’s test using IBM SPSS Statistics 20.0 (IBM Corp., Armonk, NY, USA). Graphs were generated using GraphPad Prism 8.0 (GraphPad Software, San Diego, CA, USA). *p* < 0.05 was considered statistically significant.

## 4. Conclusions

In conclusion, BHPE exerted a dual regulatory effect on intestinal glucose availability by inhibiting α-glucosidase activity and suppressing glucose transport. UPLC-QTOF-MS/MS analysis revealed that BHPE contains a complex profile of phenolic compounds, primarily consisting of anthocyanins, chlorogenic acid-related phenolic acids, and flavonoids. Enzyme kinetic, fluorescence quenching, and molecular docking studies collectively indicate that BHPE inhibits α-glucosidase through a mixed-type mechanism, with representative phenolic compounds engaging the enzyme via non-covalent interactions including hydrogen bonding and hydrophobic contacts. In a Caco-2 monolayer model, BHPE reduced cellular glucose uptake and trans-epithelial glucose transport in a concentration-dependent manner, accompanied by a significant downregulation of *SGLT1* and *GLUT2* mRNA expression. Future studies should include α-amylase inhibition assays and starch-based food matrix models to more comprehensively assess the role of BHPE in regulating complex carbohydrate digestion. In vivo studies and protein-level validation of glucose transporters are also needed to confirm the physiological relevance of these findings.

## Figures and Tables

**Table 1:** Tentative identification of phenolic compounds in blue honeysuckle polyphenol extract (BHPE) by UPLC–QTOF–MS/MS.

	No.	RT (Min)	Formula	Ion Mode	Calculated (*m*/*z*)	Measured (*m*/*z*)	Error (ppm)	MS/MS Fragment Ions (*m*/*z*)	Identification
Anthocyanins	1	1.86	C_27_H_31_O_16_	[M]+	611.1607	611.1603	−0.52	449.1075; 287.0548	Cyanidin-3,5-diglucoside
	2	2.47	C_28_H_33_O_16_	[M]+	625.1763	625.1740	−3.64	463.1226; 301.0707	Peonidin-3,5-diglucoside
	3	2.88	C_21_H_21_O_11_	[M]+	449.1078	449.1079	0.13	287.0548	Cyanidin-3-O-glucoside
	4	3.44	C_21_H_21_O_10_	[M]+	433.1129	433.1125	−0.97	271.0606	Pelargonidin-3-O-glucoside
	5	3.61	C_22_H_23_O_11_	[M]+	463.1240	463.1252	2.50	301.0709; 286.0476	Peonidin-3-O-glucoside
	6	3.77	C_28_H_33_O_15_	[M]+	609.1820	609.1817	−0.42	301.0712	Peonidin-3-O-rutinoside
	7	5.10	C_27_H_31_O_16_	[M]+	611.1607	611.1608	0.15	303.0491	Delphinidin-3-O-rutinoside
	8	5.26	C_21_H_21_O_12_	[M]+	465.1028	465.1037	2.05	303.0495	Delphinidin-3-O-glucoside
	9	5.29	C_27_H_31_O_15_	[M]+	595.1658	595.1647	−1.76	449.1080; 287.0549	Cyanidin-3-O-rutinoside
Phenolic Acids	10	1.99	C_16_H_18_O_9_	[M − H]−	353.0878	353.0880	0.64	191.0570	Neochlorogenic acid
	11	2.73	C_16_H_18_O_9_	[M − H]−	353.0878	353.0883	1.34	191.0563	Chlorogenic acid
	12	3.56	C_16_H_18_O_9_	[M − H]−	353.0878	353.0883	2.83	191.0570	Cryptochlorogenic acid
	13	5.70	C_25_H_24_O_12_	[M − H]−	515.1193	515.1193	0.00	353.0877; 191.0560	Isochlorogenic acid C
	14	5.91	C_25_H_24_O_12_	[M − H]−	515.1193	515.1193	0.00	353.0877; 191.0560	Isochlorogenic acid B
Flavonols	15	2.91	C_15_H_10_O_6_	[M + H]+	287.0550	287.0557	2.46	213.0536	Kaempferol
	16	3.14	C_27_H_30_O_15_	[M + H]+	595.1700	595.1600	−1.76	287.0600	Luteolin-7-O-rutinoside
	17	5.10	C_15_H_10_O_7_	[M + H]+	303.0499	303.0501	0.62	229.0541	Quercetin
	18	5.10	C_21_H_20_O_12_	[M + H]+	465.1028	465.1020	−1.52	303.0493	Quercetin-3-O-hexoside
	19	6.11	C_28_H_32_O_15_	[M + H]+	609.1820	609.1817	−0.42	301.0712	Diosmetin-7-neohesperidoside
	20	7.90	C_16_H_12_O_7_	[M + H]+	315.0509	315.0509	0.00	165.0202	Isorhamnetin
Flavanols	21	2.30	C_30_H_26_O_12_	[M + H]+	579.1497	579.1479	−3.07	409.0925; 271.0595	Proanthocyanidin B1
	22	2.68	C_15_H_14_O_6_	[M + H]+	291.0863	291.0884	7.12	139.0390; 123.0439	Catechin
	23	3.57	C_15_H_14_O_6_	[M + H]+	291.0863	291.0884	7.12	139.0390; 123.0439	Epicatechin
	24	4.10	C_30_H_26_O_12_	[M + H]+	579.1497	579.1497	−5.37	409.0925; 271.0595	Proanthocyanidin dimer

Note: Compounds were tentatively identified based on retention time, accurate mass, MS/MS fragmentation patterns, and comparison with published data. RT, retention time; MS/MS, tandem mass spectrometry.

**Table 2:** Fluorescence quenching and binding parameters for the interaction between BHPE and α-glucosidase at different temperatures.

T (K)	K_sv_ (10^−2^ mL/μg)	K_q_ (10^6^ mL/(μg·s))	Ka (mL/μg)	*n*
298	2.34 ± 0.12	2.34	27.40	0.91
310	1.71 ± 0.01	1.71	78.32	1.06

Note: Ksv, Stern–Volmer quenching constant; Kq, apparent quenching rate constant; Ka, apparent binding constant; *n*, number of binding sites. Constants were calculated based on the mass concentration of BHPE.

## Data Availability

Dataset available on request from the authors.

## Supplementary Materials

The following supporting information can be downloaded at: https://www.mdpi.com/article/10.3390/molecules31122146/s1, Table S1: Primers for quantitative real-time PCR.

## Abbreviations

The following abbreviations are used in this manuscript:

BHPE	Blue honeysuckle polyphenol extract
SGLT1	Sodium-dependent glucose cotransporter 1
GLUT2	Sodium-dependent glucose transporter 2
