# Supplementary material for: Blue Honeysuckle (Lonicera caerulea L.) Polyphenol Extract Inhibits α-Glucosidase Activity and Modulates Glucose Transport in Caco-2 Cells
Source: Molecules. 2026 Jun 18;31(12):2146. doi: 10.3390/molecules31122146 (PMC13304497; doi:10.3390/molecules31122146)
Supplement: Supplementary file 1 [file molecules-31-02146-s001.zip › molecules-4358343-supplementary.pdf]

Supporting Material For

Blue Honeysuckle (Lonicera caerulea L.) Polyphenol Extract Inhibits α-Glucosidase Activity and Modulates Glucose Transport in Caco-2 Cells

Table S1 Primers for quantitative real-time PCR.

Primer name	Sequences
GAPDH	Forward: 5′ - GAAAGCCTGCCGGTGACTAA - 3′
	Reverse: 5′ - AGGAAAAGCATCACCCGGAG - 3′
SGLT1	Forward: 5′ - CCATGGACAGTAGCACCTGG - 3′
	Reverse: 5′ - AGATATCGGCTGCATTGCGA - 3′
GLUT2	Forward: 5′ - GCCACACTCACACAAGACCT - 3′
	Reverse: 5′ - AACTGGAAGGAACCCAGCAC - 3′
